# An In Vivo Model of Separate *M. tuberculosis* Phagocytosis by Neutrophils and Macrophages: Gene Expression Profiles in the Parasite and Disease Development in the Mouse Host

**DOI:** 10.3390/ijms23062961

**Published:** 2022-03-09

**Authors:** Elena Kondratieva, Konstantin Majorov, Artem Grigorov, Yulia Skvortsova, Tatiana Kondratieva, Elvira Rubakova, Irina Linge, Tatyana Azhikina, Alexander Apt

**Affiliations:** 1 Laboratory for Immunogenetics, Central Research TB Institute, 107564 Moscow, Russia; alyonakondratyeva74@gmail.com (E.K.); majorov@list.ru (K.M.); tanya00747@gmail.com (T.K.); rubakova@mail.ru (E.R.); iralinge@gmail.com (I.L.); 2 Shemyakin-Ovchinnikov Institute of Bioorganic Chemistry, Russian Academy of Sciences, 117997 Moscow, Russia; artgrigorov@gmail.com (A.G.); ju.skvortsova@gmail.com (Y.S.); tatazhik@ibch.ru (T.A.)

**Keywords:** *M. tuberculosis* transcriptome, neutrophils, macrophages, infection progression, immune response, mouse model

## Abstract

The role of neutrophils in tuberculosis infection remains less well studied compared to that of the CD4^+^ T-lymphocytes and macrophages. Thus, alterations in *Mycobacterium tuberculosis* transcription profile following phagocytosis by neutrophils and how these shifts differ from those caused by macrophage phagocytosis remain unknown. We developed a mouse model that allows obtaining large amounts of either neutrophils or macrophages infected in vivo with *M. tuberculosis* for mycobacteria isolation in quantities sufficient for the whole genome RNA sequencing and aerosol challenge of mice. Here, we present: (i) the differences in transcription profiles of mycobacteria isolated from liquid cultures, neutrophils and macrophages infected in vivo; (ii) phenotypes of infection and lung inflammation (life span, colony forming units (CFU) counts in organs, lung pathology, immune cells infiltration and cytokine production) in genetically TB-susceptible mice identically infected via respiratory tract with neutrophil-passaged (NP), macrophage-passaged (MP) and conventionally prepared (CP) mycobacteria. Two-hour residence within neutrophils caused transcriptome shifts consistent with mycobacterial transition to dormancy and diminished their capacity to attract immune cells to infected lung tissue. Mycobacterial multiplication in organs did not depend upon pre-phagocytosis, whilst survival time of infected mice was shorter in the group infected with NP bacilli. We also discuss possible reasons for these phenotypic divergences.

## 1. Introduction

Neutrophils are the most commonly mycobacteria-infected phagocytes in the respiratory tract of TB patients [1], in the lungs of *M. tuberculosis*-infected genetically susceptible mice shortly after challenge [2], and the first intruders in TB lung cavities after aspiration of their caseous material and drug sanitation [3]. Nevertheless, the role of neutrophils in tuberculosis infection remains less well studied and more controversial than that of CD4^+^ T-lymphocytes and macrophages [4,5]. Systemic studies of neutrophils in TB began in the late 1990s; initial works suggested that neutrophils contribute to early defense against infection by diminishing mycobacterial CFU counts in organs [6,7]. However, when refined neutrophil-depleting antibodies were introduced and a better understanding of neutrophil interactions with other immune cells was achieved, more and more data consistent with a deleterious rather than protective role of neutrophils in lung TB inflammation accumulated [8,9,10,11,12].

The weight of evidence associating lung neutrophil influx with disease severity is impressive, but how this deleterious effect is executed remains unclear. At least two, non-mutually exclusive, hypotheses were discussed. First, activation and degranulation of neutrophils with subsequent release of high amounts of biologically active substances meant to target the offending pathogen has been long considered as an important factor of tissue damage during neutrophil inflammation (discussed by [13,14]). These considerations were supported by observations in the mouse *M. tuberculosis* and *M. avium* infection models: neutrophils form necrotic foci in infected lungs surrounded by thick layers of highly hypoxic tissue, which severely affects breathing capacity of the organ [15]. Second, there is ample evidence that neutrophils readily engulf but poorly kill virulent mycobacteria [2,16,17]. The “Trojan horse” hypothesis was proposed, suggesting that lung neutrophils may promote survival of mycobacteria by providing a temporary shelter from activated pulmonary macrophages [2]. Subsequent studies seriously questioned this simplified view, providing evidence that efferocytosis of infected apoptotic and necrotic neutrophils by macrophages and dendritic cells has profound and different consequences for the activation of acquired immunity and the outcome of infection (see [10,18,19,20,21] for the review). It became increasingly clear that, inside neutrophils, mycobacteria themselves acquire new properties and modify physiology of the host phagocytes.

There are intrinsic difficulties in working with such fragile and short-living cells as neutrophils; despite substantial recent progress in our understanding of mycobacteria-neutrophil interactions, several important issues remain unaddressed. Thus, whilst shifts in mycobacterial transcription profiles resulted from intra-macrophage persistence were characterized using microchip [22,23,24] and RNA-seq [25] approaches, analogous data for the intra-neutrophil persistence are lacking. In addition, we do not know whether the parameters of experimental infection initiated in laboratory animals by cryopreserved bacilli reactivated in cultural media—the method used practically in all in vivo studies—differ from those triggered by mycobacteria that already passed through phagocytes. In this regard, we consider important the observations made by Davis and Ramakrishnan [26] in zebra fish, indicating that mycobacterial spread through apoptotic cells is a major way of macrophage infection in vivo. Meanwhile, in mammals, a good proportion of such infected apoptotic cells would be neutrophils.

To address these important questions, we developed an experimental approach that allows for obtaining large amounts of either neutrophils or macrophages infected in vivo with virulent *M. tuberculosis*, isolating mycobacteria in quantities sufficient for the whole genome RNA sequencing and triggering TB infection in mice by aerosol challenge. Here, we describe this experimental system and its application for clarifying two issues. First, we present the differences in transcription profiles of mycobacteria isolated from conventional liquid cultures, neutrophils and macrophages infected in vivo. Second, we evaluated major phenotypes of infection and lung inflammation (life span, CFU counts in organs, lung pathology, immune cell infiltration and cytokine production) in genetically TB-susceptible I/St mice, identically infected via respiratory tract with neutrophil-passaged, macrophage-passaged and conventionally prepared mycobacteria. The goal of this second part of our work was to find out whether a short initial interaction of mycobacteria with two different major phagocyte populations leads to notable differences in the development and outcome of chronic TB infection.

## 2. Results and Discussion

### 2.1. Gene Expression Profiles in Mycobacteria

#### 2.1.1. General Picture

To determine the changes in *M. tuberculosis* transcriptome following phagocytosis, we profiled transcriptomes of neutrophil-passaged (NP), macrophage-passaged (MP) and culture-passaged (CP) bacilli. Each sample was obtained in three independent biological replicates. The statistics of RNA-seq, details of mapping sequencing results and the lists of differentially expressed genes (DEGs) are displayed in Supplementary Tables S1 and S2. In total, 456 and 379 genes were up-regulated and 344 and 284 genes were down-regulated in MP and NP mycobacteria, respectively.

#### 2.1.2. Common Gene Expression Changes for MP and NP Mycobacteria

**Figure 1:**
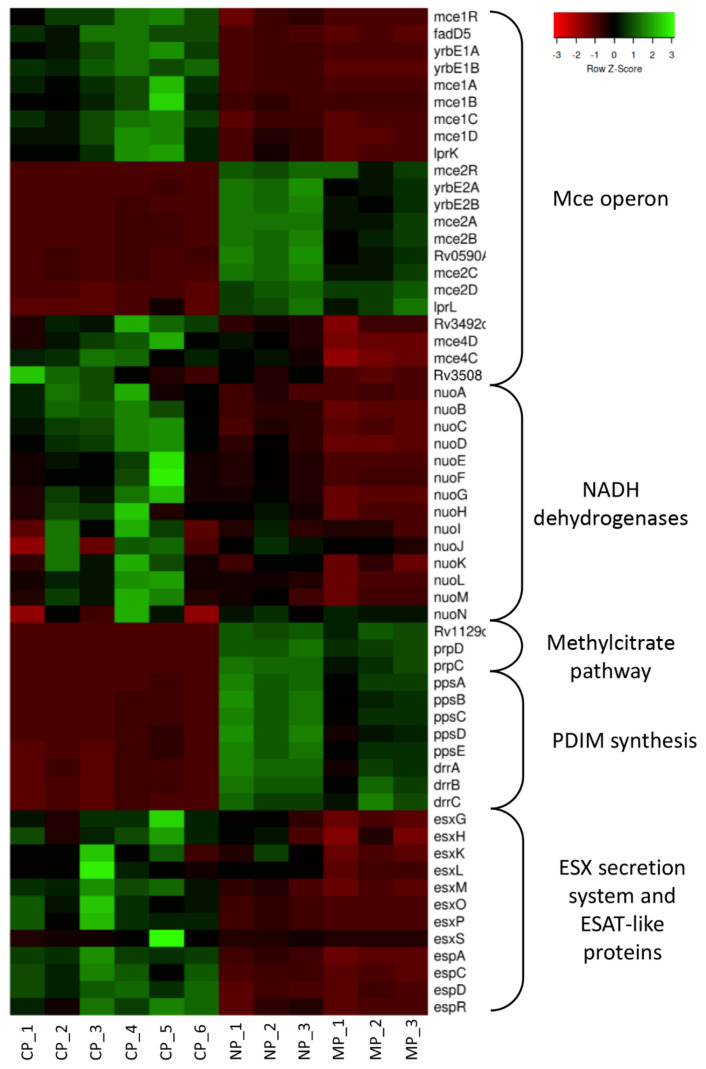
Differential RNA-seq of *M. tuberculosis* cultured in Dubos medium (CP), engulfed by neutrophils (NP) or macrophages (MP). Heat map of *M. tuberculosis* RNAs differentially expressed across individual biological replicates (*p* < 0.05; log2FC ≥ 1.5).

Superposition of transcriptomes displayed in Tables S1 and S2 allows for concluding that changes in gene expression profiles following engulfment by two major phagocyte populations are similar. The list of common DEGs comprises 236 up-regulated and 145 down-regulated genes (Table S3 and Figure 1). Clusters of functionally related *M. tuberculosis* genes with similar expression tendency after phagocytosis are depicted in Figure 1. The most prominent functional clusters are briefly described below.

(a) Increased expression of transcription regulators

Numerous corresponding genes are mostly up-regulated in MP and NP mycobacteria. The group includes many families, e.g., AraC (*Rv3833*), ArsR (*Rv2640c*, *Rv2642*), HTH-type (*kstR*, *kstR2*, *smtB, Rv1994, Rv2640c, Rv2642, Rv3744,* and *Rv2034*), MerR (*Rv3334*), TetR (*Rv3167c*, *Rv3173c*) and antitoxins (*parD1*, *relF*, *vapB12, vapB14*, vapB41, *vapB43*, *vapB7*, *vapB9*, *vapB25*). Some particular genes with established or putative functions are:

*PrpR* (*Rv1129c*), which regulates the expression of key proteins in the methylcitrate (PrpD and PrpC) and glyoxylate (isocitrate lyase Icl1) pathways [27], is up-regulated more than 100-fold.

*KstR* (*Rv3574*) transcriptional regulator, which controls the expression of genes involved in lipid catabolism [28], including another transcriptional regulator *Rv3557* from the TetR family [29], is up-regulated more than 10-fold.

*SigE* (*Rv2123*), the alternative sigma factor, is extremely important for intracellular mycobacterial survival and activates *prp* and *icl1* genes [30].

*IclR* (*Rv2989)* has multiple targets in the *M. tuberculosis* genome, was shown to activate proteins of glyoxylate shunt [31] and demonstrated 10-fold up-regulation.

*Rv1994* (*cmtR*), *Rv2640c*, *Rv2642*, *Rv3744* (*nmtR*), and *Rv2034*, the members of the ArsR-SmtB family of transcriptional regulators showed increased expression during persistence in phagocytes [32]. These genes regulate bacterial stress caused by metal deficiency [33].

*WhiB* transcription factors, *whiB7* activated during oxidative stress [34], and *whiB6* associated with the ESX-1 secretion system, which is involved in dissemination of mycobacteria and granuloma formation [35].

(b) PE/PPE proteins and ESX secretion systems

Among genes encoding proteins of the PE/PPE superfamily, the transcription levels of 18 genes differed significantly (at least 3-fold) between phagocytized and cultured mycobacteria. The functions of most of these genes are not known; however, some are clustered in *ESX* operons and are secreted through the ESX-5 secretion system [36]. Expression of genes within the *ESX-1* locus is down-regulated inside both neutrophils and macrophages. Corresponding proteins play an important role in TB pathogenesis, infection dissemination, and granuloma formation [26]. Thus, the ESAT-6, a well-known virulence factor, is secreted via ESX-1 [37,38]. ESAT-6 is also known as a membrane-lysing factor that promotes the release of mycobacteria from phagosomes [39]. Expression of ESAT-6 (*Rv3875*) dropped 4-fold in NP and 2-fold in MP compared to CP mycobacteria. The level of expression of genes for ESAT-like proteins (*esxB*, *C*, *D*, *I*, *J*, *L*, *O*, *P*) also decreased following phagocytosis.

(c) Mammalian cell entry (*Mce*) operons

The expression level of genes from the *Mce2* operon in NP and MP bacteria was 5–40-fold higher, whilst that of *Mce1* and *Mce4* operons was 5–10-fold lower, compared to control. The functions of corresponding proteins have not been fully elucidated, but it is known that they belong to the transport systems necessary for interaction of mycobacteria with host cells [40]. It was shown recently that the *Mce1* and *Mce4* operons encode systems of lipid transportation across the cell wall [41]. Diacyltrehaloses (DAT), polyacyltrehaloses (PAT) and phthiocerol dimycocerosates (PDIM) production depend upon *Mce1* operon expression. The proteins encoded by the *Mce4* operon are thought to be involved in cholesterol catabolism [42], whilst Mce2 proteins are involved in metabolism and import of sulfolipids [43].

(d) PDIM synthesis

PDIM cell wall glycolipid is one of the most important virulence factors involved in macrophage infection and resistance of mycobacteria to NO stress [44,45]. PDIM levels directly correlate with *M. tuberculosis* escape from phagosomes and induction of host cell necrosis, leading to mycobacterial dissemination [46]. Genes involved in PDIM synthesis (*fadD26*, *ppsA-E*, *drrA-B*, *papA5*) and their transcriptional regulator *Rv3167c* are up-regulated both in NP and MP bacteria compared to control level.

#### 2.1.3. Mycobacterial Genes Differentially Changing the Expression within Macrophages and Neutrophils

**Figure 2:**
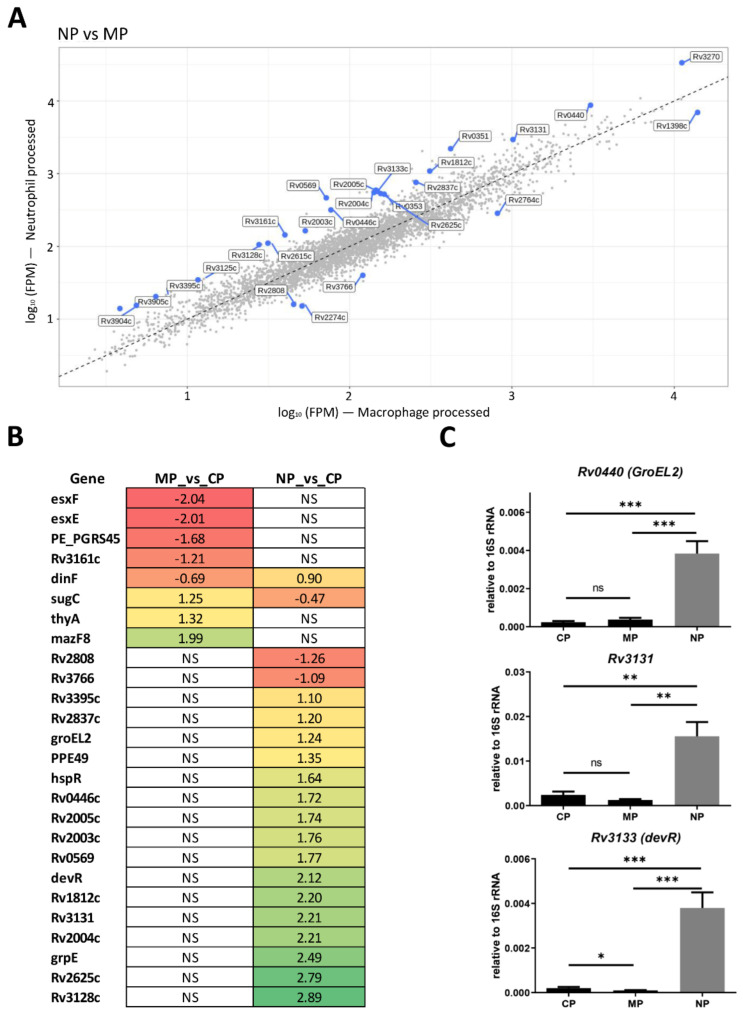
*M. tuberculosis* genes differentially expressed after phagocytosis by macrophages (MP) and neutrophils (NP). (**A**) Scatterplot comparing transcript measurements (FPM, fragments per million mapped) for MP and NP transcriptomes. Each dot represents one transcript. Genes that are differentially expressed in MP and NP are marked by blue dots. Scatter plot built using ViDGER package [54]. (**B**) Heat map of DEGs. Heat map was visualized by TPM (transcripts per kilobase million) approach using heat-mapper web tool [55]. The log_2_FC numbers of genes differentially expressed in MP and NP versus CP are provided; NS—non significant. (**C**) Validation of DEGs by qRT-PCR. mRNA expression levels of *Rv0440*, *Rv3131*, and *Rv3133* genes were estimated in MP, NP, and CP cDNA samples. mRNA expression was normalized to that of 16S rRNA. Statistical analysis was performed using GraphPad Prism 6.0 (GraphPad Software Inc., La Jolla, CA). The data are presented as the mean ± SD of three biological replicates for each sample, Mann–Whitney U-test. Differences were considered statistically significant at *p* < 0.05. * *p* < 0.05, ** < 0.01, and *** < 0.001, respectively.

Despite a high level of similarity between gene expression profiles of NP and MP mycobacteria, a number of genes demonstrated different changes of expression in two types of phagocytes (Table S4, Figure 2A,B). Compared to the CP control, the expression of *Rv3905c* and *Rv3904c* genes encoding ESAT-6-like proteins EsxF and EsxE, *Rv2615c* encoding a protein from the PE-PGRS family, and *Rv3161c* encoding dioxygenase, was down-regulated in MP but not in NP bacteria. Recently, it was shown that EsxE and EsxF are essential for the tuberculosis necrotizing toxin (TNT) secretion and suggested that the pore-forming activity of the EsxE-EsxF complex may increase membrane permeability of phagosomes, thus, facilitating mycobacterial escape from the host cell [47]. The expression of *Rv2764c* encoding ThyA thymidylate synthase and Rv2274c encoding MazF8 toxin was increased in MP but did not change in NP cells.

Interestingly, more genes differed from controls regarding their expression in NP than in MP group. Thus, 16 genes were up-regulated more then 3-fold, including 9 genes belonging to the *Dos* regulon: *Rv0569*, *Rv1812c*, *Rv2003c*, *Rv2004c*, *Rv2005c*, *Rv2625c*, *Rv3128c*, *Rv3131* and their transcriptional regulator *Rv3133* (*DosR* or *DevR)* itself. Genes from the *Dos* regulon are known to be activated under hypoxia and NO-induced stress and are involved in the establishment and maintenance of reduced metabolism during dormancy [48]. In addition, proteins encoded by *Rv2004c*, *Rv2005c*, and *Rv3131* are immunogenic and stimulate production of pro-inflammatory cytokines via the TLR2 signaling pathway [49]. Two other genes with unknown functions, *Rv2808* and *Rv3766*, were down-regulated.

Another group of genes selectively increasing expression in neutrophils encodes chaperone proteins GrpE (HSP70 cofactor), GroEL2, and their transcriptional regulator HspR. Chaperones Hsp70 and GroEL2 also function as adhesins [50]. A shortened form of GroEL2 in mycobacteria is involved in blocking apoptosis in macrophages [51]. Exposure of macrophages to GroEL2 interferes with MHC-II expression and T-lymphocyte activation, suggesting its role in *M. tuberculosis* survival within the host cells [52].

To independently confirm sequencing results, the expression levels of GroEL2-encoding *Rv0440*, *Rv3131,* and DosR-encoding *Rv3133* genes were determined by quantitative RT-PCR. The results showed concordance with the RNA-seq data (Figure 2C).

Overall, the transcriptional reprogramming after phagocytosis includes a marked increase in the expression levels of transcriptional regulators involved in the methylcitrate and glyoxylate pathways, which is typical for mycobacterial persistence in vivo. Accumulating propionyl-CoA may be incorporated into various cell wall components, such as PDIM and sulfolipid-1, especially given that the expression of genes involved in PDIM synthesis is also increased. On the other hand, engulfed mycobacteria inactivate ESX secretion systems, especially ESX-1. Among transcriptome differences between MP and NP mycobacteria, it is worth mentioning prominent up-regulation of several *DosR* regulon genes, including their regulator *DosR*, and genes for GrpE/GroEL2 chaperons and their transcriptional regulator *HspR* in the NP group. This pattern is consistent with reduced metabolic activity of mycobacteria and transition to dormancy [48,53].

### 2.2. TB Infection and Immune Response

#### 2.2.1. Lung Infiltration by Immune Cells and Cytokine Production

**Figure 3:**
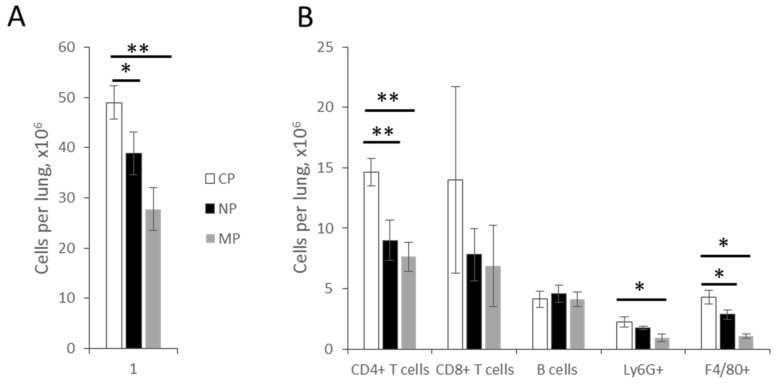
Analysis of cell numbers in lungs of I/St mice 8 weeks post infection by culture-passaged (CP), neutrophil-passaged (NP) or macrophage-passaged (MP) M. tuberculosis. Total numbers (**A**) and FACS analysis of main populations (**B**) of cells counted per right lung. Five mice in each group were assessed individually and the results presented as mean ± SD, * *p* < 0.05, ** < 0.01, ANOVA.

**Figure A1:**
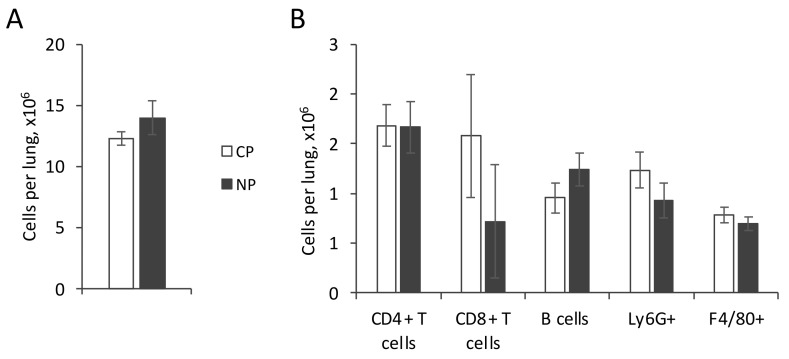
Lung cell numbers in I/St mice 3 weeks post infection with culture-passaged (CP) or neutrophil-passaged (NP) *M. tuberculosis.* Total numbers (**A**) and FACS analysis of main immune cell populations (**B**) counted per right lung.

To find out how the shifts in gene expression profiles described above affect mycobacterial immunogenicity and virulence, we infected TB-susceptible I/St mice with identical doses of NP, MP and CP mycobacteria. We first evaluated the difference between mycobacterial capacity to attract immune cells to the lung tissue at two key time points of adaptive immunity formation after aerosol challenge: establishment of T-cell response by week 3 and its plateauing after week 4 post-challenge [15,56]. No differences in total lung cellularity and immune cell mobilization were found between mice infected with mycobacteria from culture or from infected neutrophils at week 3 post-challenge (Figure A1 (See Appendix A)), However, by week 8 of infection, i.e., at the stage of a balance between adaptive immune response and infection progression, mycobacteria passaged either through neutrophils or macrophages induced a more mild general lung infiltration (cellularity) compared to their counterparts cultured in vitro (Figure 3). Moreover, differences in lung-infiltrating immune cells profiles compared to CP controls were very similar for NP- and MP-infected groups: these mice display significantly lower infiltration with CD4^+^ T-cells, F4/80^+^ macrophages and Ly6G^+^ neutrophils, whereas the content of CD19^+^ B-lymphocytes and CD8^+^ T-lymphocytes was equal in all three groups (Figure 3). Whilst the reason for a delay in the expression of phenotypic differences remains unknown, a decrease in inflammatory capacity of NP and MP mycobacteria corresponds well to the nature of shifts in their gene expression profiles after phagocytosis. It is likely that mycobacteria show a bias toward dormant, less immunogenic phenotypes due to a decreased expression of genes encoding secreted immunodominant virulence factors. Regarding this, our hypothesis that a short residence within neutrophils increases mycobacterial pathogenicity is not supported by cytological characteristics of lung inflammation.

#### 2.2.2. Mycobacterial Multiplication and Disease Severity

**Figure 4:**
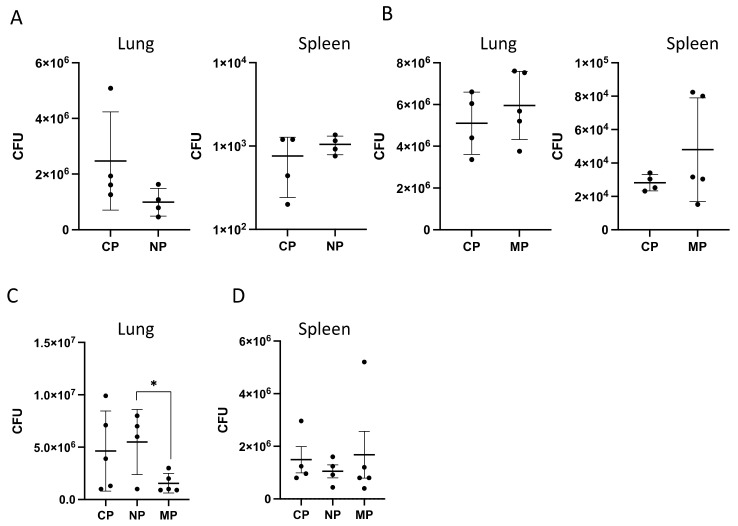
Mycobacterial CFU counts in organs at the stages of infection corresponding to establishment (**A**,**B**) and advanced (**C**,**D**) stages of adaptive immune response. Mice in groups of 4–5 were infected via respiratory tract with indicated types of mycobacteria. Results of one of two similar experiments are displayed as mean CFU ± SD per the right lung. * *p* < 0.05, ANOVA. Results of the second experiment are displayed in Figure 3A.

**Figure 5:**
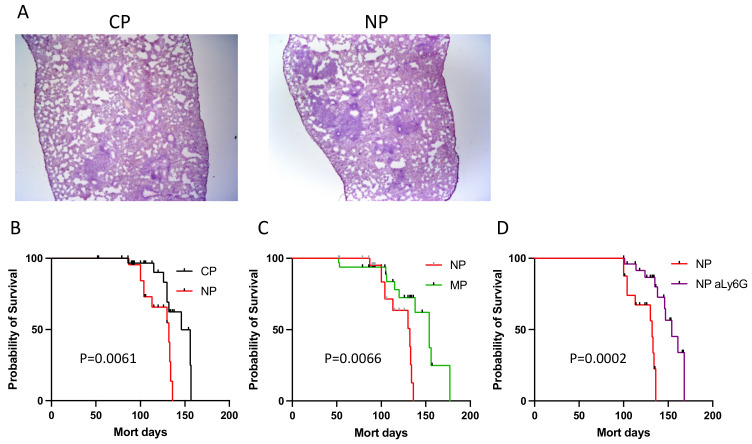
Lung pathology in mice infected with NP mycobacteria (**A**) and survival time of infected mice (**B**–**D**). (**A**) At week 4 post-challenge, mice infected with CP and NP mycobacteria developed condensed inflammatory foci in the lungs surrounded by zones of non-inflamed, porous breathing tissue. (**B**) NP- and CP-infected, (**C**) NP- and MP-infected, (**D**) NP-infected neutrophil-depleted and control animals. Mice in groups of 8–10 were infected with ~80 mycobacteria from indicated stocks. Survival was monitored daily starting day 50 post-challenge and survival curves compared using log-rank (Mantel-Cox) test.

In parallel, we assessed whether mice infected with NP, MP and CP mycobacteria develop different patterns of disease progression. To this end, we assessed three classical phenotypes that characterize progression of TB infection—mycobacterial multiplication in lungs and spleens, development of lung pathology and survival time post-challenge. We found that at the early stages of anti-mycobacterial adaptive immunity (weeks 3 and 4) groups of mice did not differ regarding mycobacterial growth in organs (Figure 4A,B). However, at week 8, multiplication of MP bacteria residing in the lungs was significantly reduced compared to that of CP and NP (Figure 4C left panel), whereas disseminated splenic populations did not differ in sizes (Figure 4C right panel). The differences in immune cell infiltration of the lung between mice infected with previously phagocytized mycobacteria and control mice (Figure 3) were not accompanied by clear differences in the degree of lung pathology in the groups (Figure 5A, *p* > 0.05 for the zones of condensed inflammation, ANOVA).

The most striking difference in disease phenotypes induced by mycobacteria of different origin was a significantly diminished survival time of the group infected by NP bacilli compare to the CP and MP groups (Figure 5B,C). Given the lack of differences between pictures of lung pathology and lung CFU counts between NP and control groups, this result suggested that a short transition of mycobacteria through neutrophils elevated either their virulence or their survival within the host cells without multiplication. Taking into account that the shift in gene expression profile of NP mycobacteria corresponds exactly to dormancy transition, the latter possibility looked more likely, especially since the life span is the phenotype with the latest expression.

However, there was a possibility that, within a relatively short time post infection all mycobacteria, irrespective to their interactions with primary phagocytes, undergo several rounds of phagocytosis by host cells vigorously migrating toward developing TB focus and engulfing mycobacteria released from dying cells infected earlier, which may interfere with reliable interpretation of the results. Importantly, in I/St mice, this early recruited phagocyte population is predominantly comprised of neutrophils [2]. Thus, we performed experiments on in vivo neutrophil depletion with highly specific anti-Ly-6G antibodies (clone 1A8) at days −1 and +1 of infection to abrogate, for a few days, the possibility of mycobacterial capture by freshly arrived neutrophils, as demonstrated in our earlier study [11].

Temporary neutrophil depletion in vivo significantly prolonged survival of NP-infected mice (Figure 5D), confirming deleterious effect of neutrophil circulation in vivo at the early phase of TB infection. Combined with the results displayed in Figure 5B,C, this consistent pattern suggests that rapid changes in the gene expression profile of mycobacteria within infected neutrophils are sufficient to increase their harmful influence on the disease outcome. Thus, mycobacterial switch to a less active metabolic state paradoxically leads to a more rapid death of the host.

What are the possible reasons for a delayed phenotype expression and mismatches between parameters of infection described above? We suggest that rapid shifts in gene expression profiles following phagocytosis reflect a general picture of what happens with the whole mycobacterial and host cells populations, leaving the question about the events that occur in individual infected host cells or TB foci unanswered. In other words, it looks likely that a substantial proportion of mycobacterial population indeed transits to dormancy, which results in their prolonged survival within the host, but a part of the population preserves its virulence and induces progressive TB infection. It is well established that primary (arising from inhaled bacilli) and secondary (arising from hematogenous re-seeding of the lung) TB granulomata differ with regard to the sizes, cytokine profiles, cellular compositions and degrees of necrotization [57,58]. Given that the secondary M. tuberculosis seeding of the lung tissue from lymphoid organs in mice occurs at day 16–17 post-challenge [59], we assume that, as early as week 3 post-challenge, we could observe a complex picture of mycobacteria-host cell interactions reflecting a mixture of primary and secondary responses. Consider this, the recently modeled and analyzed heterogeneity within neutrophil populations during TB infection [60] deserves specific attention. Bearing in mind that both mycobacterial and phagocyte populations contain cells expressing a wide spectrum of metabolic activity at each and every time point, the phenotypes we are assessing often seem to demonstrate pure stochastic diversity, but, in reality, represent superposition of metabolic activity of individual cells at different stages of infection progression.

Our present technical capacities and our level of knowledge interfere with a reliable dissection of such systems. However, this will be the subject of studies in the near future, and the first issue to be improved is the way we trigger the infection itself. A “standard” dose for TB modeling in mice (50–100 CFU per mouse by aerosol route) is 20–30-fold higher than that initiating infection in humans (1–5 mycobacteria in a micro-droplet of coughed-out sputum). Infection with “standard” doses immediately creates multiple foci in the lung tissue, each providing profoundly different environment. Thus, we evaluate complex disease parameters using some resultant phenotypes rather than assess contribution of an individual infection focus. The problem of independent TB pathogenesis in individual granuloma was already discussed in detail [58], and several animal models of TB infection triggered by the aerosol administration of 1–3 mycobacteria were established [61,62]. Although these approaches are costly and time-consuming, their adequacy, compared to conventional models, looks obvious. Combination of ultra-low challenging doses with investigation of individual infectious foci by single cell RNA sequencing methods should result in a better understanding of molecular TB pathogenesis.

## 3. Materials and Methods

### 3.1. Gene Expression Profiling

*RNA Isolation*. RNA was extracted from cultured mycobacterial cell and mycobacteria engulfed by neutrophil and macrophages by phenol-chloroform extraction. Cells were pre-destructed with 0.1 mm zirconium beads (BioSpec Products, Bartlesville, OK, USA) as described [63]. RNA samples were treated with DNAase I (Invitrogen, Carlsbad, CA, USA) to remove contaminating genomic DNA. The amount and purity of RNA were determined on the NanoDrop 2000C spectrophotometer.

*Libraries for RNA-Seq and Data Analyses*. RNA samples were depleted of rRNA using the Ribo-Zero Epidemiology reagent kit (Epicentre, Madison, WI) and were used for generating sequencing libraries with the NEBNext Ultra II Directional RNA Library Prep Kit (New England Biolabs, NEB), according to the manufacturers’ protocol. Sequencing was performed in triplicates using the Illumina NovaSeq6000 as the pair-ended 100 nt reads. After quality control evaluation, the reads were mapped on the reference *M. tuberculosis* H37Rv genome (GenBank accession number AL123456.3) by Bowtie2 [64]; statistical data processing was performed using Pearl scripts. Differential expression analysis was performed using the DESeq2 software package [65]. Genes were considered differentially expressed at *p*-values < 0.05, FDR ≤ 0.1, log_2_FC (fold change) ≥ 1.5. Functional categories and metabolic pathways were assigned using TubercuList [66] and PATRIC databases [67]. All RNA-seq data generated for this study has been deposited in the GEO repository under accession numbers GSE140156 and GSE158465.

*cDNA Synthesis and qRT-PCR*. cDNA was synthesized from 1 mg of total RNA using random hexanucleotides and SuperScript III reverse transcriptase (Life Technologies, Carlsbad, CA) according to the manufacturer’s protocol. qRT-PCR was performed with specific primers (Supplementary Table S5) and qPCRmix-HS SYBR mix (Evrogen, Moscow, Russia) in a LightCycler 480 Real-Time PCR system (Roche, Basel, Switzerland) at the following cycling conditions: 95 °C for 20 s, 61 °C for 20 s, and 72 °C for 30 s, all repeated 40 times. Three biological and nine technical replicates were used to ensure reproducibility; 16S rRNA expression was used for normalization. The results were analyzed with LinRegPCR v 2014.6 [68].

### 3.2. Infection and Immunity

*Mice* of the I/St strain were bred under conventional, non-specific pathogen free (non-SPF) conditions at the Animal Facilities of the Central Institute for Tuberculosis (Moscow, Russia), in accordance with the guidelines from the Russian Ministry of Health # 755, US Office of Laboratory Animal Welfare (OLAW) Assurance #A5502-11. Water and food were provided ad labium. Female mice 10–12 weeks of age at the beginning of experiments were used. All experimental procedures were approved by the Institutional Animal Care and Use Committee (IACUC), protocols 2, 3, 7, 10 of 6 March 2019.

**Figure 6:**
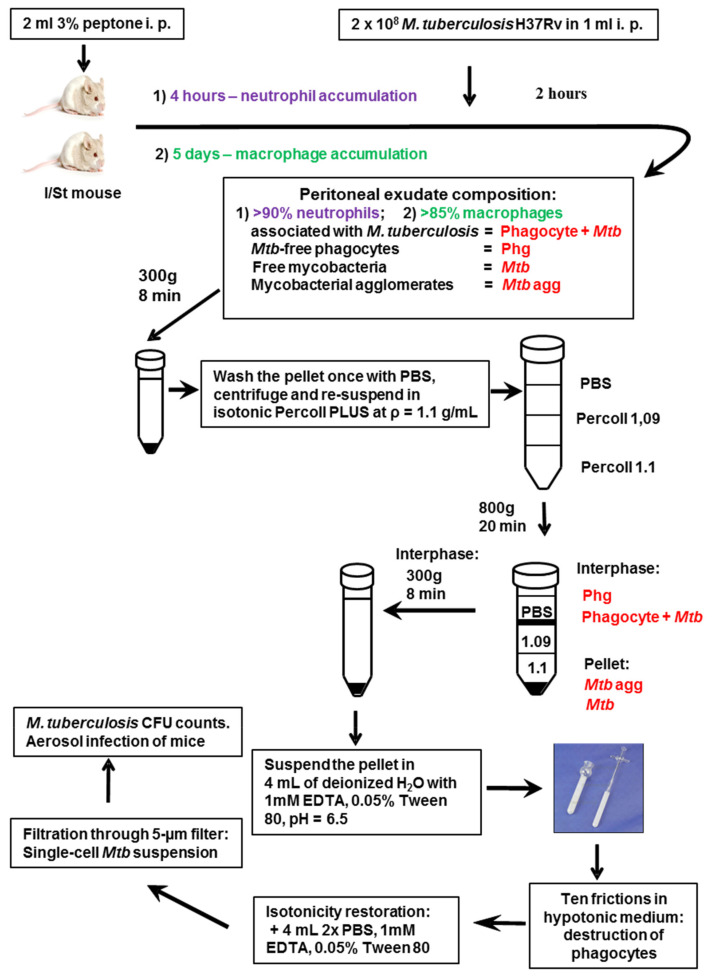
The approach for obtaining large amounts of ex vivo neutrophils or macrophages containing engulfed mycobacteria and avoiding contamination with bacilli that escaped phagocytosis (modification from Ref. [66] with permission). The layer of Percoll with ρ = 1.1 serves as an additional protector isolating the interphase from pellet.

*Phagocytes and mycobacteria extraction*. We developed the approach for obtaining large amounts of ex vivo neutrophils or macrophages containing engulfed mycobacteria and avoiding contamination with bacilli that escaped phagocytosis. For the gene expression analysis mycobacterial RNA was immediately extracted from peritoneal phagocytes centrifuged at 300× *g*. Mycobacterial isolation and CFU counting was required for in vivo experiments. The essence of our experimental procedure presented in Figure 6 is the formation of a pellet comprised of non-engulfed mycobacteria and an interphase layer of phagocytes above Percoll with ρ = 1.09 g/cm^3^, with subsequent phagocyte’s lysis and preparation of bacterial suspensions (short communication in Russian [69]). To compare infectious processes triggered by mycobacteria from different sources, it was mandatory to infect animals with identical doses of bacteria.

*Infection*. Totally, eleven independent experiments on challenging mice with NP, MP and SP mycobacteria were performed, the first five were based upon equalizing the size of inoculum by direct counting of either individual bacteria freshly isolated from phagocytes (cell cytometer, ×300 magnification) or by the micro-colony method described earlier [70]. However, assessment of lung mycobacterial counts 24 h after aerosol administration (initial uptake) constantly provided 3–4-fold higher counts for NP/MP compared to CP mycobacteria, suggesting that pre-phagocytosis itself changed association or penetration capacities of mycobacteria regarding host lung cells. Thus, for the next series of experiments, we used the following approach. NP or MP mycobacteria were applied via respiratory tract using Glas-Col aerosol chamber (Terre Haute, IN) exactly as described earlier [71] and 24 h later 4 mice from each group were sacrificed for the initial uptake measurement. Three weeks later, when the actual mycobacterial uptake was established by CFU counts on Dubos agar (Difco, Sparks, MD), the control group of mice was infected with CP bacteria whose dose was calculated as the concentration required to prepare an 8-mL suspension for aerosol formation in the machine nebulizer providing the required size of inoculum. This approach constantly resulted in a less than 20% variation in the initial mycobacterial uptake between groups and allowed us to seed 80–100 mycobacteria per mouse in each experiment.

*CFU counts, survival time and lung pathology*. At indicated time points following infection, spleens and identical lobes of the right lungs from individual mice were homogenized in 2.0 mL of sterile saline, and 10-fold serial dilutions of 0.2 mL samples were plated on Dubos agar and incubated at 37 °C for 20–22 days before CFU were counted. Survival time was monitored daily starting on day 50 post-infection. Left lungs were frozen in the regimen of −60 °C to −20 °C temperature gradient in the electronic Cryotome^®^ (ThermoShandon, UK), and serial 6–8 µm-thick sections were made across the widest area. Sections that were fixed with ice-cold acetone and stained were examined by the experienced pathologist (EK) and photographed using Axioskop40 microscope and AxioCamMRc 5 camera (Carl Zeiss, Berlin, Germany).

*Lung cell suspensions*. Lungs were enzymatically digested as described previously [2,72]. Briefly, blood vessels were washed out by heart perfusion via vena cava cut with 0.02% EDTA-PBS; lungs were removed, sliced into 1–2 mm^3^ pieces and incubated at 37 °C for 90 min in supplemented RPMI-1640 containing 200 U/mL collagenase and 50 U/mL DNase-I (Sigma, St. Louis, MO, USA). Single cell suspensions from 5 mice were obtained individually, washed twice in HBSS containing 2% FCS and antibiotics. A total of 3 × 10^5^ cells were used for assessment of surface phenotypes and the remaining sample was used for cell culturing and cytokine assessment.

*Cell phenotypes* were analyzed by flow cytometry using the FACS Calibur machine (BD Biosciences). The following labeled monoclonal antibodies were used: CD4-PerCp (dilution 1:300), CD4-AF488 (dilution 1:200), or CD4-PE (dilution 1:200), CD19-AF647 (6D5, dilution 1:400), F4/80-AF-488 (BM8)—all from Biolegend, Germany; Ly6G-PE (1A8, BD Biosciences, dilution 1:200). Results are presented as mean cell number per lobe ± SEM.

*Cell cultures and cytokine ELISA*. A total of 1 × 10^6^ lung cells from each mouse were cultured overnight in the presence of a 10 µg/mL mixture of mycobacterial antigens (cultural filtrate, CF) in supplemented RPMI-1640 medium in wells of 24-well plates for 48 h. CF was obtained by culturing *M. tuberculosis* as a biofilm on the surface of the protein-free Sauton’s medium for 4 weeks, followed by precipitation of mycobacteria at 3000 g for 20 min. The protein content in the liquid phase was concentrated using an Amicon cell device and was brought to the final 1mg/mL protein with sterile saline. Cytokine contents in supernatants were assessed in the ELISA format using ELISA MAX kits (Biolegend, Germany) for IL-6, IL-10, IFN-γ and TNF-α according to manufacturer’s instructions.

*Statistics*. Statistical analysis was performed using GraphPad Prism 6.0 (GraphPad Software Inc., La Jolla, CA, USA). Application of ANOVA, Mann–Whitney and log-rank Mantel–Cox tests is indicated in figure legends. References to statistical methods of gene expression profiles evaluation are provided in the legend to Figure 2.

## Figures and Tables

**Figure A2:**
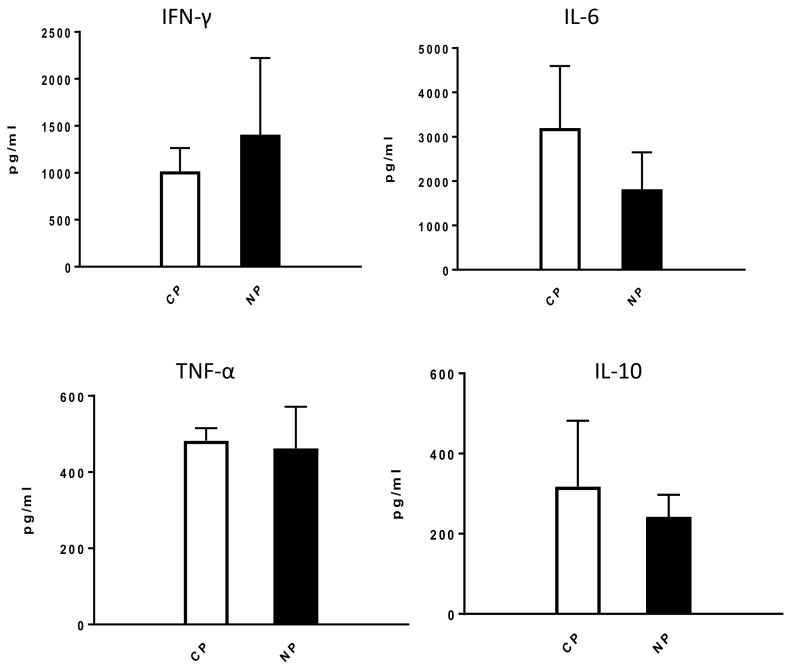
Production of major regulatory cytokines by lung cells incubated for 48 h with mycobacterial antigens. ELISA assessment of lung cell supernatants. 5 mice per group, *p* > 0.05 for all cytokines, ANOVA.

**Figure A3:**
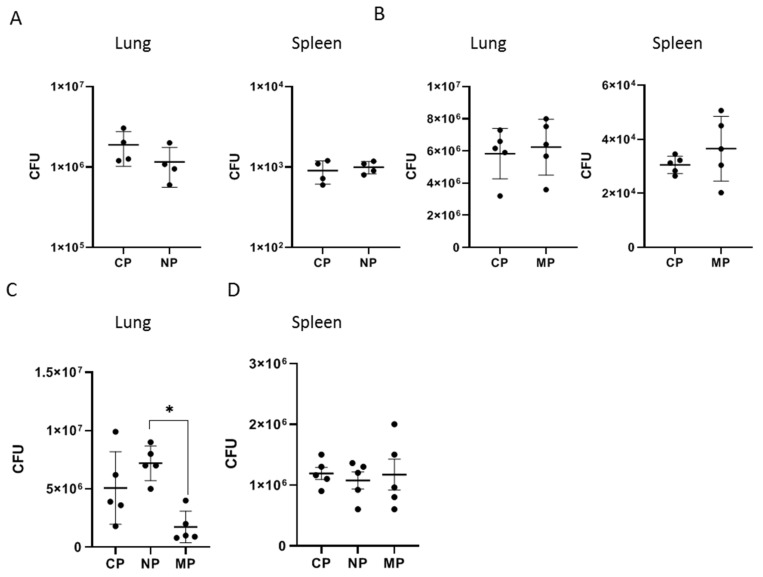
Mycobacterial CFU counts in organs at the stages of infection corresponding to establishment (**A**,**B**) and advanced (**C**,**D**) stages of adaptive immune response. Results of the second experiment are displayed as mean CFU ± SD per the right lung. * *p* < 0.05, ANOVA.

## Data Availability

All RNA-seq data generated for this study has been deposited in the GEO repository under accession numbers GSE140156 and GSE158465.

## Supplementary Materials

The following supporting information can be downloaded at: https://www.mdpi.com/article/10.3390/ijms23062961/s1.
