# Supplementary material for: An In Vivo Model of Separate M. tuberculosis Phagocytosis by Neutrophils and Macrophages: Gene Expression Profiles in the Parasite and Disease Development in the Mouse Host
Source: Int J Mol Sci. 2022 Mar 9;23(6):2961. doi: 10.3390/ijms23062961 (PMC8954342; doi:10.3390/ijms23062961)
Supplement: Supplementary file 1 [file ijms-23-02961-s001.zip › Supplementary Table 5.pdf]

Supplementary Table S1. Oligonucleotides used for qPCR analysis

Name	Sequence
Rv3131_qfor	GGG GTA CAA CCG CTT GTT CCC
Rv3131_qrev	GGT CTA GGG CAC TGA CCT GC
Rv0440_qfor	CAA GGC GAT GCT GCA GGA TAT
Rv0440_qrev	GGT GGT CTC GTC CTT GGT GAC
16S_qfor	TACGTAGGGTGCGAGCGTTG
16S-qrev	CCCGCACGCTCACAGTTAAG
DosR-qfor	TGTCGCGGTGCTGGATGTC
DosR-qrev	ATTCCCTTGATGTCTTTGACGAC
